# Age- and sex-specific effects of maternal separation on the acoustic startle reflex in rats: early baseline enhancement in females and blunted response to ambiguous threat

**DOI:** 10.3389/fnbeh.2022.1023513

**Published:** 2022-10-26

**Authors:** Lauren Granata, Abigail Parakoyi, Heather C. Brenhouse

**Affiliations:** Psychology Department, Northeastern University, Boston, MA, United States

**Keywords:** early life adversity (ELA), development, sex, rat, acoustic startle, ultrasonic vocalization (USV)

## Abstract

Early life adversity (ELA) increases the incidence of later-life anxiety disorders. Dysregulated threat processing, including responsivity to ambiguous threats, is an indicator of anxiety disorders and can be influenced by childhood experiences. The acoustic startle response is a defensive reflex displayed by mammals when exposed to sudden intense stimuli reflecting individual variations in vigilance. These measures can be altered by previous experience and experimental modifications, including the introduction of unconditioned aversive stimuli. Rats emit ultrasonic vocalizations (USVs) in the 22 KHz range in negative contexts. As such, 22 KHz USVs are an ethologically relevant social cue of environmental threat shown to induce anxiety-like behavior in recipient rats. Because the timing of symptom manifestation after early life adversity can differ between sexes, the current study sought to identify the age- and sex-specific effects of daily maternal separation (MS) on responsivity to ambiguous threat in rats. In Experiment 1, rat pups underwent MS or control rearing from postnatal day (P) 2–20, then underwent behavioral testing beginning on P24, 34, or 54 to determine whether MS modified the baseline startle response or the modulation of startle by 22 KHz USVs. In Experiment 2, rats were tested in a light-enhanced startle paradigm at P54 after MS or control rearing to determine whether MS influenced light-enhanced startle. Results show an enhancement of the baseline startle magnitude by MS in females at P34. At P54, MS reduced the modulation of the startle response by 22 KHz USVs and prevented light-enhanced startle, indicating an MS-induced deficit in defensive responsivity when exposed to potential threat.

## Introduction

Exposure to adversity early in life often leads to detrimental affective phenotypes that involve heightened responsivity to potential threats (Edmiston and Blackford, 2013; Silvers et al., 2016). Because early life adversity (ELA)-related disorders often first emerge in adolescence (Kessler et al., 2001; Andersen and Teicher, 2008; Davey et al., 2008), intervening variables found in clinical studies make the role that ELA plays in these diseases difficult to interpret. Moreover, the challenging nature of developmental studies has mired the ability to uncover the mechanisms underpinning discernible sex differences in ELA-attributable vulnerability. In fact, ELA yields an earlier age of onset for anxiety and depression, with more robust effects in girls than in boys (Marcus et al., 2005; Rudolph and Flynn, 2007; Schuch et al., 2014; Weeks et al., 2014).

The acoustic startle reflex is an evolutionarily conserved defensive behavioral response to sudden and intense stimuli (Koch and Schnitzler, 1997). In rodents, the reflex is demonstrated by a contraction of the head and neck muscles, measurable by the latency to onset, latency to peak response, magnitude, and duration. Exaggerated responses on these outcome measures are indicative of increased arousal and hypervigilance. ELA has been associated with either enhanced or blunted startle responses depending on whether outcomes were measured in a neutral context or with emotionally-valenced stimuli (Jovanovic et al., 2009; Klauke et al., 2012; Quevedo et al., 2015). Heightened startle at baseline, a measure of physiological arousal associated with trait anxiety (Beck and Catuzzi, 2013), is found in adults with a history of childhood trauma (Jovanovic et al., 2009; Klauke et al., 2012). We know of no studies to date that directly investigated sex differences in acoustic startle following ELA, however, there are mixed reports of sex differences in post-traumatic stress disorder-related acoustic startle (Beck and Catuzzi, 2013), likely due to wide varieties in the type of trauma exposure.

Affective picture viewing studies in humans have demonstrated that the startle reflex is modulated by emotional valence, such that, relative to neutral pictures, startle is potentiated when viewing unpleasant pictures that depict a possible threat and attenuated when viewing pleasant pictures (Lang et al., 1990). Potentiated startle in the presence of uncertain threat is a core feature of several internalizing psychopathologies (Gorka et al., 2017), and is a reliable measure of corticolimbic-driven threat sensitivity in both rodents and humans (Davis et al., 2010; Thome et al., 2018). In humans, viewing or anticipating images of fearful faces, in particular, has been shown to induce potentiation of the startle reflex, whereas neutral and happy faces do not facilitate the same response (Anokhin and Golosheykin, 2010). Compared to other affective pictures, facial expressions produce a greater autonomic response and more pronounced activation of brain regions involved in processing emotionally-valenced stimuli, like the amygdala (Hariri et al., 2002). This response to fearful faces is positively correlated with the subjective experience of anxiety, indicating that the degree to which startle is enhanced reflects changing emotional states. However, adults who experienced ELA display a diminished startle response during anticipation of such aversive stimuli (Stout et al., 2021). Suppressed potentiation of startle can result from coordinated efforts of peripheral and central inhibitory mechanisms that control threat responsivity and develop differently after ELA, namely pro-inflammatory immune signaling (Beck and Catuzzi, 2013; Brenhouse, 2022), Hypothalamic-pituitary-adrenal (HPA)-derived glucocorticoid signaling (Lee et al., 1994), and corticolimbic regulation between the amygdala and anterior cingulate (Pissiota et al., 2003).

After experiencing ELA, the onset of psychiatric disturbance is protracted (Kessler et al., 2001; Andersen and Teicher, 2008; Davey et al., 2008), and symptom onset may differ between males and females. This is particularly relevant during the adolescent transition, which is characterized by increased reactivity, risk-taking, and emotional sensitivity (Spear, 2000). It is therefore important to track the development of symptomatology to identify sex-specific vulnerabilities and windows for intervention. In humans without ELA exposure, there is evidence that startle potentiation by aversive stimuli emerges during adolescence, but this trajectory could be offset by early childhood neglect (Quevedo et al., 2015). Few studies in humans have directly compared individuals on the basis of sex, but differing patterns of results have been found between studies examining gender-homogenous populations (Medina et al., 2001; Costanzo et al., 2016). This demonstrates the need to investigate sex-specific effects of ELA on potentiated startle, and further, to directly compare males and females.

Traditional paradigms in animals to assess potential threat responsivity have limitations (Molendijk and de Kloet, 2015; Demin et al., 2019), due to the lack of translatability or relevance to human experience. Importantly, childhood maltreatment-evoked anxiety in humans largely involves sensitivity to social threats (Sandre et al., 2018), as measured in fearful-face paradigms (Dannlowski et al., 2012). Here, we assessed ELA-associated changes in responsiveness to a socially relevant, emotionally-valenced stimulus in rats (Demaestri et al., 2019). Specifically, responsivity to recordings of threatening ultrasonic vocalizations (USV) emitted by a conspecific was used to determine sex-specific changes to threat sensitivity in ELA-exposed rats. USVs in the 22  KHz range are typically emitted by rats as early as juvenility when in anxiety- or fear-provoking situations, while 55  KHz range USVs are typically emitted in appetitive situations (Burgdorf et al., 2008; Wöhr et al., 2016). We have previously shown that 22 Hz USV presentation can elicit anxiety-like and defensive behaviors while activating neurons in regions underlying threat detection and responsiveness, such as the basolateral amygdala (BLA) and bed nucleus of the stria terminalis (BNST; Demaestri et al., 2019). Similarly, others have found enhanced startle magnitude in response to 22 KHz USV playback, but these experiments were only carried out in male rats (Inagaki and Ushida, 2017). Moreover, the effects of ELA on the startle response during exposure to 22 KHz USVs have not been investigated in other ages. A widely used assessment of startle modulation in anxiogenic contexts is the light-enhanced startle test (de Jongh et al., 2002). Sustained exposure to a bright light, a non-social aversive stimulus, enhances the startle response (Davis et al., 1997; de Jongh et al., 2002), but ELA was found to prevent light-enhanced startle in adult female, but not male, rats (de Jongh et al., 2005). Because the current study sought to determine the effects of ELA on startle in response to 22 KHz USVs, we also conducted a light-enhanced startle experiment in order to obviate potential unknown sex-specific responses to USV stimuli.

Maternal separation (MS) is an ELA paradigm in which pups are separated from their dam and littermates for several hours per day from postnatal day (P) 2–20. MS has been shown to disrupt the mother-infant relationship and is an experimental model for the study of childhood neglect (Lehmann and Feldon, 2000; Walker and McCormick, 2009; Brenhouse and Bath, 2019). MS has been described as an environment of deprivation and, to a lesser extent, threat (McLaughlin et al., 2014), and we have recently reported that MS confers an unpredictable environment as well (manuscript under review). Adversity within the dimensions of threat and unpredictability has particularly been associated with anxiety-like behaviors due to functional and developmental impairments of connectivity between regions such as the BLA, BNST, and prefrontal cortex (PFC; Tottenham et al., 2010; Hein and Monk, 2017; Spadoni et al., 2022). With a developmental study in males and females, we have observed that MS yields hyperinnervation from the basolateral amygdala to the prefrontal cortex, with females, in particular, displaying this hyperinnervation earlier in development (as early as P25), along with disrupted maturation of BLA-medial PFC functional connectivity (Honeycutt et al., 2020). Therefore, we hypothesized that if MS disrupts the development of threat responsive circuits that regulate acoustic startle, with females impacted earlier than males, then MS exposure will enhance baseline threat sensitivity earlier in females. We further hypothesized that if the circuitry controlling affective regulation of startle is disrupted following MS, MS will blunt a typical potentiation of startle in the presence of 22 KHz USV playback or light, with an exploratory investigation of when during development effects on USV potentiation would emerge.

## Methods

### Animals

All experiments were performed in accordance with the 1996 Guide for the Care and Use of Laboratory Animals (NIH) with approval from the Institutional Animal Care and Use Committee at Northeastern University. Animals were housed under standard laboratory conditions in polycarbonate wire-top caged with pine shave bedding, a plexiglass tube for enrichment, and food and water available *ad libitum*. The facility was kept on a 12-h light/dark cycle (light period between 07:00 and 19:00) with regulated temperature (22–23°C) and humidity (37%–53%). Throughout all procedures, animals were left undisturbed except for weekly cage cleanings and to take pup weights on postnatal day (P) 9 and P20.

### Maternal separation

Male and female Sprague-Dawley rats originally obtained from Charles River Laboratories (Wilmington, MA) were mated in-house. One male and one female were caged together until pregnancy was confirmed by checking for the presence of sperm in a vaginal swab each morning for a maximum of 4 days. All females were primiparous, and pregnant dams were housed singly upon confirmation of pregnancy. Parturition was checked daily, and the day of birth was denoted as P0. Each litter was randomly assigned to maternal separation (MS) or control (Con) treatment conditions. On P1, litters were culled to 10 pups with five males and five females when possible. Pup sex was determined by anogenital distance on P1.

MS litters underwent separations daily from P2 to P20. From P2 to P10, each pup was placed in an individual plastic cup containing pine shavings from the home cage to maintain a familiar odor. Cups were placed in a circulating water bath kept at 37°C for 3.5 h (09:30–13:0 h). From P11 to P20, when pups are able to adequately thermoregulate, each pup was placed in an individual mouse cage containing clean bedding mixed with a small handful of home cage bedding for 4 h (09:30–13:30). MS dams remained in the home cage in a separate room for the duration of the separation.

Subjects were identified by toe clips, which were performed on P5. Con litters were left undisturbed except for normal husbandry procedures, including toe clips on P5 and weights on P9 and P20. We have previously reported (Grassi-Oliveira et al., 2016), and consistently observe, that MS animals gain less weight during the MS paradigm than Con animals but that MS and Con animals are equal in weight at P25, P35, and P55. To control for the experimenter interaction received by MS pups, Con pups were also briefly handled by an experimenter on P12 and P15 for 3 min each. On P21, all rats were weaned and pair-housed in standard caging with sex- and condition-matched cage mates. No more than two male and two females per litter were used in each experimental group.

### Acoustic startle test

Experiments 1 and 2 used the acoustic startle hardware and software package from Med Associates (Med Associates product number: MED-ASR-PRO1). The startle cabinets were equipped with sound attenuating foam on all walls and doors. Each cabinet held a grid rod animal holder on a startle platform containing the load cell. The load cell and load cell amplifier were used to convert force on the platform to a voltage representing the startle response. Speakers for delivering white noise background and startle noise bursts were positioned 1” behind the animal holder. The grids rods on the back of the animal holder provide ventilation and do not interfere with sounds. Four different chambers were counterbalanced between sexes and rearing groups.

### Experiment 1a: effects of MS on development of the acoustic startle response

**Figure 1:**
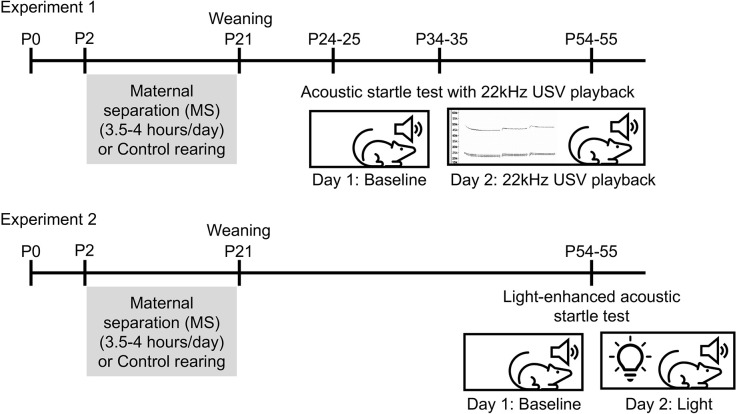
Experimental timelines. In Experiment 1, rats underwent maternal separation (MS) or control rearing from P2 to 20 until weaning on P21. Separate cohorts of rats underwent the acoustic startle test with 22 KHz USV playback on either P24–25, P34–35, or P54–55. In Experiment 2, rats underwent MS from P2 to 20 and were tested in light-enhanced startle on P54–55.

Experimental design is illustrated in Figure 1. Con and MS rats were tested beginning on P24 (10 Con males, 11 MS males, 10 Con females, 10 MS females), P34 (11 Con males, nine MS males, 10 Con females, 10 MS females), or P54 (eight Con males, 11 MS males, 10 Con females, 10 MS females) in a 2-day paradigm. Different animals were used for each age, so no animal was tested more than once. Because the rats’ weights differ between ages and sexes, the startle boxes were calibrated to accommodate the weight of each rat being tested. The purpose of Day 1 was to establish a baseline startle response for each animal. On Day 1, rats were transported to the testing room (~50 lux) and left to acclimate for 10 min. After acclimation, each rat was placed into the animal holder in the unlit startle cabinet. The experiment began with 5 min of white background noise to acclimate the animal to the startle cabinet. Then, 100 stimulus tones were presented at 30-s intervals. Each stimulus was a 50 ms white noise tone of 95 dB, 100 dB, 105 dB, or 110 dB presented in random order with a 3 ms rise/fall time. The background noise level was set to 70 dB throughout the experiment. At the end of 100 trials, rats were removed from the boxes and returned to their home cages. Boxes were cleaned with 40% EtOH between runs.

### Experiment 1b: effects of MS on 22 KHz USV-modulated acoustic startle

The purpose of Day 2 was to determine the rats’ response to startle stimulus tones after being exposed to a 22 KHz USV, a social cue signifying potential threat. Twenty-four hours after the Day 1 test, rats were transported to the testing room and left to acclimate for 10 min. Similar to Day 1, rats were placed in the animal holders, and the experiment began with a 5-min acclimation period. Then, rats were presented with a 5-min 22 KHz USV recording previously obtained from an adult male rat exposed to cat urine. The USV was recorded using a condenser ultrasonic microphone (Avisoft-Bioacoustics CM16/CMPA; frequency range 2–200 KHz), and played back using a D/A converter/power amplifier and ultrasonic speaker (Avisoft-Bioacoustics USG Player 416H, Vifa speakers) situated inside the startle cabinet 1” behind the animal holder. After the USV playback, rats were presented with 30 stimulus tones at 30-s intervals. Stimulus tones were 50 ms in duration, 95 dB, 100 dB, 105 dB, or 110 dB in volume, and presented in random order with a 3 ms rise/fall time. At the end of Day 2, rats were removed from the boxes and returned to their home cages.

### Experiment 2: effects of MS on light-enhanced acoustic startle

After observing the effects of USV in the first cohort of P55 animals, we aimed to test responses to a different anxiogenic context. Con and MS rats (11 Con males, 10 MS males, 10 Con females, nine MS females) were tested beginning on P54 in a 2-day light-enhanced startle paradigm. Startle boxes were calibrated to accommodate the weight of each rat. On Day 1, rats were transported to the testing room and allowed 10 min of acclimation to the room. After the acclimation period, each rat was placed in the calibrated animal holder in the startle cabinet. The experiment began with a 5-min acclimation followed by 30 stimulus tones presented at 30-s intervals. Each tone was a 50 ms 105 dB white noise burst with a 3 ms rise/fall time. Because the baseline data from Experiment 1 separated by stimulus intensity showed that the 95 dB stimulus did not reliably generate a strong startle response (data not shown), and to avoid ceiling effects at 110 dB, Experiment 2 presented stimulus tones only at 105 dB. The background noise level was set to 70 dB throughout the experiment. At the end of the 30 trials, rats were removed from the boxes and returned to their home cages. Boxes were cleaned with 40% EtOH between runs.

Day 2 of the experiment aimed to determine the rats’ response to stimulus tones while exposed to light, a naturally aversive stimulus. Notably, the illumination was chosen for moderate intensity in order to provide a diffusely, rather than acutely, threatening environment. Twenty-four hours after the Day 1 test, rats were transported to the experimentation room and allowed to acclimate for 10 min. Rats were then placed in the calibrated animal holder in the startle cabinet. A wireless LED puck light located 4 inches behind the animal holder inside each startle cabinet was turned on before the start of the experiment (~70 lux). Rats underwent the same experimental protocol as Day 1 (5-min acclimation to background white noise followed by 30 105 dB stimulus tones). At the end of Day 2, rats were returned to their home cages.

### Statistical analysis

Outcome measures represent the average across all trials on Day 1 or Day 2 for each subject. For baseline measures, we report the latency to onset (ms), latency to peak (ms), peak startle value (arbitrary units), and duration (ms), described in Table 1. Total and average startle magnitudes during the startle period were also analyzed, but data are not shown because results were redundant to the results of the peak value. The response to USV or light presentation was represented by the subject’s Day 2 value as a percentage of the Day 1 value for each outcome measure. Statistical tests were performed in jamovi software (The jamovi project, Version 1.6). To determine the effects of MS and sex on the baseline startle response and the percent change from baseline after USV or light presentation, two-way ANOVAs were performed for each outcome measure. Effects were considered significant when *p* < 0.05. When there was a significant interaction between rearing condition and sex, Tukey’s *post hoc* comparisons were performed to determine group differences between males and females.

**Table 1:** Descriptions of outcome measures from the acoustic startle test.

**Dependent variable**	**Definition**
**Latency to onset (ms)**	Time between white noise burst onset and response onset
**Latency to peak (ms)**	Time between white noise burst onset and response peak
**Peak value (arbitrary units)**	Greatest startle amplitude in the response window
**Duration (ms)**	Time between response onset and end of response

## Results

### Experiment 1a: effects of MS on the baseline startle response on P24, 34, and 54

**Figure 2:**
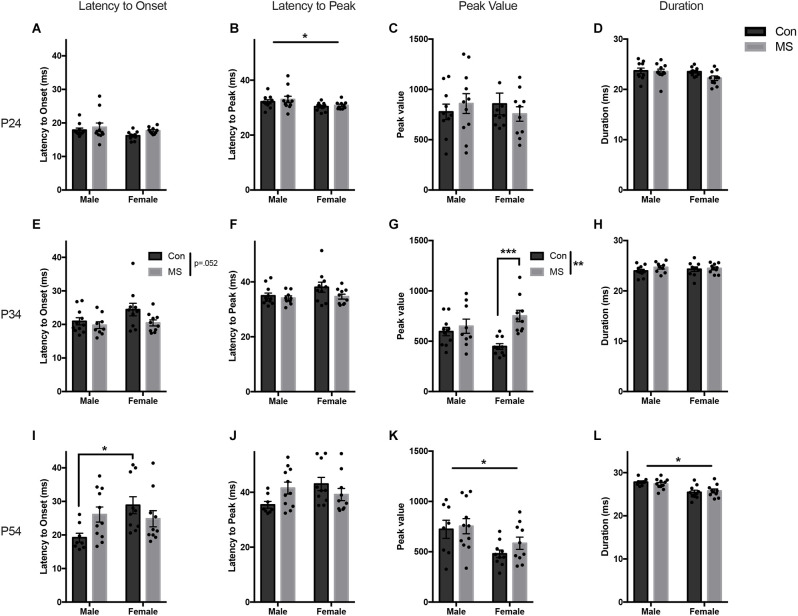
Results from Experiment 1a, the effects of maternal separation (MS) on the baseline startle response. Graphs depict the latency to onset (ms), latency to peak (ms), peak value (arbitrary units), and duration (ms) on P24 **(A–D)**, P34 **(E–H)**, and P54 **(I–L)**. Control-reared groups are depicted by dark gray bars and MS-reared groups are depicted by light gray bars. Figures represent the mean group values ±SEM. Individual subjects are depicted by data points. *p < 0.05, **p < 0.01, ***p < 0.001.

All statistics for experiment 1a can be found in Supplementary Table 1. In P24 animals at baseline, there were no effects of sex, rearing, or sex by rearing interaction on the latency to onset, peak value, or duration (Figures 2A,C,D). Females had a shorter latency to peak than males (*F*_(1,37)_ = 6.1045, *p* = 0.018, $\eta_{p}^{2}$ = 0.142), but there were no effects of rearing or sex by rearing interaction on latency to peak (Figure 2B).

At P34, there were no effects of sex, rearing, or sex by rearing interaction on latency to onset, latency to peak, or duration (Figures 2E,F,H). The magnitude of the startle response was greater in MS animals, demonstrated by increased peak value (*F*_(1,36)_ = 12.476, *p* = 0.001, $\eta_{p}^{2}$ = 0.257), and there was a sex by rearing interaction (*F*_(1,36)_ = 6.163, *p* = 0.018, $\eta_{p}^{2}$ = 0.146; Figure 2G). *Post hoc* comparisons revealed significant increases in peak startle magnitude in MS females compared to Con females (*t* = −4.264, *p* < 0.001, *d* = −1.907), and no difference in males (*t* = −0.74, *p* = 0.88, *d* = −0.333).

A sex difference was observed at P54, with males startling at a greater magnitude and for longer than females (peak value: *F*_(1,35)_ = 9.394, *p* = 0.004, $\eta_{p}^{2}$ = 0.212: duration: *F*_(1,35)_ = 23.1727, *p* < 0.017, $\eta_{p}^{2}$ = 0.398; Figures 2K,L). There were no main effects of rearing condition on any startle outcome measure. However, there was a sex by rearing interaction on latency to onset (*F*_(1,35)_ = 5.734, *p* = 0.022, $\eta_{p}^{2}$ = 0.141; Figure 2I) and latency to peak (*F*_(1,35)_ = 5.304, *p* < 0.027, $\eta_{p}^{2}$ = 0.132; Figure 2J). *Post hoc* comparisons revealed that Con males had a shorter latency to onset than Con females (*t* = −2.883, *p* = 0.032, *d* = −1.367), but MS males and females did not differ (*t* = 0.405, *p* < 0.977, *d* = 0.177). No group-wise differences were found in the latency to peak.

### Experiment 1b: effects of MS on 22 KHz USV-modulated startle

**Figure 3:**
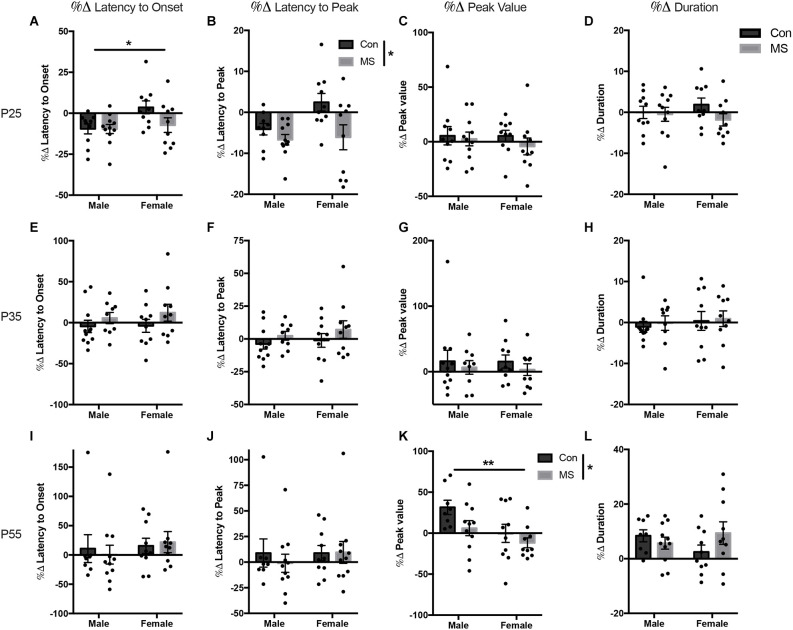
Results from Experiment 1b, the effects of maternal separation (MS) on the change in startle response from baseline after exposure to 22 KHz USVs. Graphs depict the change in each measure as a percentage of the value from the baseline test. Percent change from baseline in latency to onset, latency to peak, peak value, and duration on P24 **(A–D)**, P34 **(E–H)**, and P54 **(I–L)**. Control-reared groups are depicted by dark gray bars and MS-reared groups are depicted by light gray bars. Figures represent the mean group values ±SEM. Individual subjects are depicted by data points. *p < 0.05, **p < 0.01.

All statistics for experiment 1b can be found in Supplementary Table 2. At P25, there was a main effect of sex on the change in the latency to onset, with males decreasing their latency to onset more than females (*F*_(1,37)_ = 4.66, *p* = 0.037, $\eta_{p}^{2}$ = 0.112; Figure 3A), but there was no effect of rearing or sex by rearing interaction. There was a main effect of rearing on the change in latency to peak, with MS animals displaying a decrease in latency to peak compared to Con animals (*F*_(1,37)_ = 7.01, *p* = 0.012, $\eta_{p}^{2}$ = 0.163; Figure 3B). There was no effect of sex, rearing, or sex by rearing interactions in the change in peak value or duration after USV presentation (Figures 3C,D). At P35, the change in startle response from baseline after USV presentation was not affected by sex or rearing on any outcome measure (Figures 3E–H). At P55, MS reduced the change in startle magnitude from baseline after USV presentation, blunting the enhancement of startle by USV presentation (%Δ peak value: *F*_(1,35)_ = 4.127, *p* = 0.050, $\eta_{p}^{2}$ = 0.132; Figure 3K). Additionally, females displayed reduced change from baseline compared to males (%Δ peak value: *F*_(1,35)_ = 7.469, *p* = 0.010, $\eta_{p}^{2}$ = 0.176). There was no sex by rearing interaction in peak value change. There were no rearing, sex, or rearing by sex interactions in the change in latency to onset, latency to peak, or duration at P55 (Figures 3I,J,L).

### Experiment 2: effects of MS on P55 light-enhanced startle

**Figure 4:**
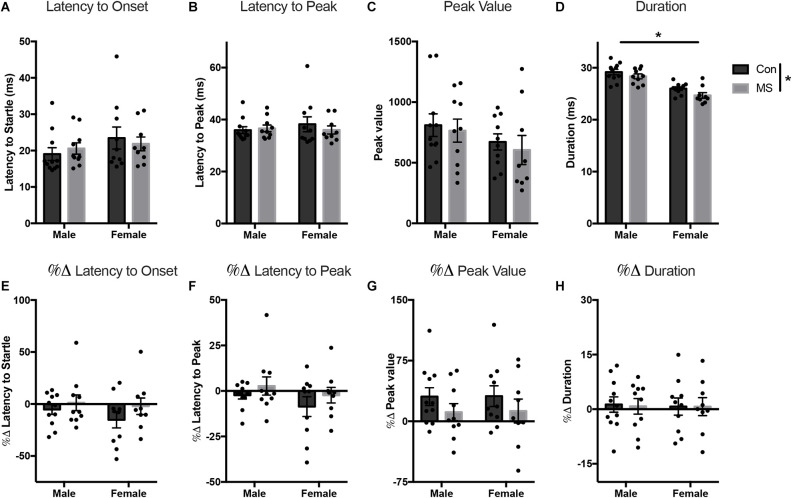
Results from Experiment 2. Effects of MS on baseline and light-enhanced startle on P54–55. Graphs depict the latency to onset (ms) **(A)**, latency to peak (ms) **(B)**, peak value (arbitrary units) **(C)**, and duration (ms) **(D)** at baseline and the percent change from baseline in latency to onset **(E)**, latency to peak **(F)**, peak value **(G)**, and duration **(H)** during exposure to light. Control-reared groups are depicted by dark gray bars and MS-reared groups are depicted by light gray bars. Figures represent the mean group values ±SEM. Individual subjects are depicted by data points. *p < 0.05.

All statistics for Experiment 2 can be found in Supplementary Tables 3, 4. At baseline, there were no effects of sex, rearing, or sex by rearing interactions on the latency to onset, latency to peak, or peak value (Figures 4A–C). However, the duration of startle in females was lower than in males (main effect of sex: *F*_(1,36)_ = 51.801, *p* < 0.001, $\eta_{p}^{2}$ = 0.590), and duration was also shorter in MS animals compared to Con (main effect of rearing: *F*_(1,36)_ = 5.025, *p* = 0.031, $\eta_{p}^{2}$ = 0.122; Figure 4D). In the light-enhanced experiment, there were no sex- or rearing-dependent differences in the change from baseline on any startle measure (Figures 4E–H). In order to confirm replication of previously reported light-enhanced startle using brighter illumination, we performed a repeated measures ANOVA (full results in Supplementary Table 5) with between-subjects factors of sex, and rearing, and peak values from the no-light test (baseline) and the light test as repeated measures to determine whether there was an effect of light exposure. Results show a main effect of light exposure (*F*_(1,36)_ = 6.096, *p* = 0.018, $\eta_{p}^{2}$ = 0.145) and a light by rearing interaction (*F*_(1,36)_ = 4.943, *p* = 0.033, $\eta_{p}^{2}$ = 0.121). In Con animals only, there was a difference between the no-light test and the lighted test (Tukey’s *post hoc*, Con/no light vs. Con/light: *t* = −3.405, *p* = 0.009).

## Discussion

The purpose of this study was to establish sex-dependent effects of MS on the acoustic startle response over development and to characterize the effects of two distinct aversive sensory stimuli, 22 KHz USVs, and light exposure. As hypothesized, MS affected females earlier in development than males, enhancing the baseline response in young adolescent females. In older adolescents, MS changed the modulation of startle by 22 KHz USV playback and light exposure, preventing potentiation.

In Experiment 1a, we investigated age-dependent and sex-dependent effects of MS on the baseline startle response. In P24 animals, MS did not impact the baseline startle response, but females exhibited a shorter latency to peak than in males regardless of rearing condition, which aligns with another study that showed shorter latencies in P25 female rats (Boctor and Ferguson, 2009). Latency is typically negatively correlated with startle amplitude, increasing amplitude with shorter latencies (Pilz and Schnitzler, 1996), but we did not observe a sex difference in peak startle amplitude at this age. After 22 KHz USV playback on P25, latency to peak was shortened compared to baseline in Con animals, and MS further exaggerated this change from baseline. Shorter latency to peak generally reflects increased alertness and is associated with other behaviors congruent with hypervigilance (Lebow et al., 2012). Interestingly, at this age, the MS-related change in responsivity became apparent only after exposure to ambiguous threat. Latency to peak includes the time from the onset of the acoustic pulse to the peak of the motor reaction. Therefore, the measure includes both the neural delay encompassing the delay to startle, and the mechanical delay between the onset and peak of the startle. Latency to startle onset decreases with age and continues to mature through the third postnatal week of life. In weanling rats, louder stimuli begin to elicit shorter latencies to respond, demonstrating maturation of the ventral cochlear nucleus (Sheets et al., 1988). As startle delay is dependent on the three-synaptic startle reflex arc between the cochlear nucleus, caudal pontine reticular nucleus (PnC), and motor nuclei (Koch, 1999), and startle duration (time from startle onset to peak) relies on motor and neuromuscular activity (Sipos et al., 2001), it is possible that early sex differences in the baseline response may reflect sex differences in the maturation of the brainstem circuitry, downstream motor pathways, or musculature.

The effects of MS on the baseline startle response emerged in early adolescents on P34, with females displaying elevated peak value if they had undergone MS. Heightened acoustic startle at baseline is reflective of hypervigilance and increased arousal. This enhanced responsiveness is phenotypic of patients with panic disorder, PTSD, and obsessive-compulsive disorder (Grillon et al., 1994; Morgan et al., 1995; Kumari et al., 2001). As such, the baseline acoustic startle response is considered an operationalization of hypervigilance observable in a range of anxiety disorders (Seiglie et al., 2019) and is a sensitive measure of individual differences in reactivity. Human research has found that adolescents at high-risk for anxiety disorders due to parental history of such conditions showed gender-specific changes in baseline and fear-potentiated startle (Grillon et al., 1997, 1998). Girls, but not boys, in the high-risk group had a heightened startle response at baseline compared to low-risk girls, but no differences in their state of anxiety were found at the time of testing (Grillon et al., 1998). Therefore, increased acoustic startle at baseline may be an early indicator of premorbid vulnerability to anxiety disorders, and the gender difference may reflect unique developmental susceptibilities to generational anxiety. In rats, the sex-specific effect of MS on the startle response in adolescents has not been extensively studied. Previous studies have found adolescent females to be more susceptible to anxiety-like behavior after MS than male rats using other assays measuring defensiveness and hypervigilance (Jin et al., 2018; Tschetter et al., 2021). Similar effects have been seen in depressive-like behavior, specifically an earlier onset of depressive-like behavior after early life adversity in female adolescents compared to males (Goodwill et al., 2019). Notably, since female rats’ pubertal maturation occurs earlier than males (Grassi-Oliveira et al., 2016) and we did not assess pubertal status in this study, there may have been more females than in males who had initiated puberty by P34. Therefore pubertal hormones may be a factor in the observed sex-specific effects of MS.

MS-induced startle enhancement in young adolescent females may be driven by sex-specific changes in underlying circuits sensitive to MS. The baseline startle response is mediated by Corticotropin-releasing factor (CRF) receptors in the brain, and CRF-enhanced startle is dependent on the BNST (Lee and Davis, 1997). MS has been shown to exert changes in CRF mRNA expression throughout the brain, including the central amygdala (CeA), BNST, medial PFC, locus coeruleus, and paraventricular nucleus of the hypothalamus (Plotsky and Meaney, 1993; Plotsky et al., 2005), all regions involved in the defensive response (LeDoux and Pine, 2016). Although adolescent-specific alterations are less extensively studied, one study found increased CRF mRNA in the PVN of adolescent female rats after MS, but no changes in the BNST or CeA, and no changes in males (Tschetter et al., 2021). Female adolescents exposed to MS demonstrate earlier manifestation of excitatory/inhibitory imbalances in the PFC, driven by reduced expression of GABAergic markers and hyperinnervation from the amygdala (Honeycutt et al., 2020; Ohta et al., 2020). Previous work from our lab has also identified accelerated maturation of PFC innervation from the BLA in females, and females sustained this hyperinnervation through later adolescence (Honeycutt et al., 2020). These may play a role in the female-specific enhancement of acoustic startle after MS.

In P54 animals, there was no effect of MS on baseline startle, but males had a greater startle magnitude and a longer response than females. It is typical for male rodents to have a larger startle response than females, likely due to their higher body weight, muscle mass, and motor strength (Lehmann et al., 1999; Plappert et al., 2005). In response to the 22 KHz USV, MS decreased the degree to which the peak value was changed from baseline, and females had a smaller change in response than males. Con males demonstrated startle enhancement after USV playback, while females did not clearly show a potentiation or inhibition. Overall, enhancement of the startle response to 22 KHz USVs was attenuated by MS.

Aversive USVs have been used to elicit anxiogenic responses in both male and female adults (Demaestri et al., 2019; Wöhr et al., 2020). Twenty-two KHz calls are emitted during social aggression, fear learning with foot shock, cocaine and opiate withdrawal, and predator exposure (Brudzynski, 2005). As a signal of potential danger in the environment, they can elicit either behavioral inhibition or hyperlocomotion in the recipient depending on the context of the alarm call and the strain of the recipient (Beckett et al., 1997). Previous work has found increased anxiety-like behavior in the elevated zero maze and reduced locomotion when exposed to 22 KHz USVs (Demaestri et al., 2019), while others showed that alarm calls caused more escape behavior in the open field (Beckett et al., 1997; Neophytou et al., 2000). In these studies, behavioral inhibition and fleeing were both accompanied by increased c-fos activation in the basolateral amygdala. Activation of other subregions of the amygdala, thalamus, hypothalamus, and periaqueductal gray was concomitant with fleeing behavior after USV exposure (Beckett et al., 1997; Wöhr et al., 2020). Efferent pathways from these regions project to the PnC of the primary acoustic startle reflex circuit, which can enhance or inhibit the startle reflex through direct connections to motor nuclei (Davis et al., 1997; Koch, 1999; Walker et al., 2003). Startle inhibition is modulated by midbrain nuclei, including the substantia nigra reticulata, pedunculopontine tegmentum, and laterodorsal tegmental area, which receive inputs from the prefrontal cortex, amygdala, and BNST (Davis, 2006). The attenuation of the USV-modulated startle seen in the present study after MS may be caused by changes in the developmental programming of forebrain regions, directly and indirectly, affecting the startle circuit. The central amygdala has been implicated as a major modulator of classically conditioned fear-potentiated startle, while the BNST and BLA have both been shown to regulate light-enhanced startle (Beck and Catuzzi, 2013). It is not currently known whether the BLA-PFC pathway, which we have previously shown to be developmentally and sex-specifically affected by MS, underlies the attenuation of the USV-modulated startle, but these circuits will be important targets for future testing.

Importantly, nearly all of the USV playback research has been performed on male recipient rats with male rat stimulus vocalizations, despite the evidence that male and female rats respond differently to alarm calls, and their own vocalizations have different qualities and tendencies of emission (Boulanger-Bertolus and Mouly, 2021; Lenell et al., 2021). One study found reduced locomotion in male and female rats exposed to alarm calls, but females retained this behavioral inhibition even after the stimulus ended (Wöhr et al., 2020). In our paradigm, the USV playback occurs just before the startle stimulus trials began so the observed startle would not be in direct response to the USV itself. If females remain more behaviorally inhibited after USV exposure, this could explain the failure to potentiate in females.

In Experiment 2, Con animals were potentiated by exposure to light, but MS animals failed to enhance startle in this aversive context. A previous study similarly found that MS blunted light-enhanced startle, but this finding was exclusive to females (de Jongh et al., 2005). This differs from the present results, which do not demonstrate a sex-specific effect. Differences in the experimental paradigms, such as stimuli amplitude and time between testing sessions, could account for disparate findings. In the present study, startle stimuli were all 105 dB, as opposed to the 95–105 dB stimuli used in the previous study. Furthermore, the previous study used Wistar rats and an MS procedure from P2 to P14, while here we used Sprague Dawley rats and MS lasted from P2 to P20. Different strains respond to acoustic startle differently at baseline, in demonstration of prepulse inhibition, and in response to drug exposure (Buuse, 2004). Here, Experiment 1 provided the basis for using 105 dB stimuli to produce sufficient and consistent startle amplitudes, but strain differences could be attributable to distinct hearing capabilities and responses to specific stimuli, which should be considered when designing and comparing experiments.

Blunted startle potentiation in response to aversive stimuli is consistent with recent human studies showing that early childhood adversity caused reduced startle responses in negative contexts. One study found that men who experienced childhood trauma had weaker startle responses compared to men with no history of trauma specifically during anticipation of negative stimuli (Stout et al., 2021). In another study, women who suffered from PTSD due to childhood abuse demonstrated reduced startle in the presence of a danger cue, and this association was strongest in individuals with more severe clinical symptoms representing avoidance (Lis et al., 2020).

## Conclusion

The present study found intriguing sex differences in the effects of MS on the acoustic startle response depending on age and type of aversive stimulus exposure. Females were affected by MS earlier than males, demonstrating a more exaggerated defensive response at baseline in early adolescence. Interestingly, 22 KHz USV-enhancement was only found in control males later in adolescence, although MS decreased the change in startle response from baseline after exposure to USVs regardless of sex. This differed from the light-enhanced experiment, in which Con animals displayed potentiation by light regardless of sex, but MS animals failed to potentiate in the presence of light. This demonstrates that males and females may respond differently to specific aversive stimuli, and different sensory modalities should be further explored. Overall, the present experiments demonstrate sex- and age-specific deficits in defensive responsiveness after MS, specifically by preventing ethologically relevant adaptive responses to potential threat.

## Data Availability Statement

The raw data supporting the conclusions of this article will be made available by the authors, without undue reservation.

## Ethics Statement

The animal study was reviewed and approved by Northeastern University IACUC.

## Author Contributions

LG, AP, and HB contributed to writing and editing of the manuscript. LG and HB designed the studies. LG and AP collected data for the studies. HB contributed funding for the study and supervised all stages of data collection, analysis, and interpretation. All authors contributed to the article and approved the submitted version.

## Funding

This work was funded by 1R01MH127850-01.
