# Supplementary material for: Age- and sex-specific effects of maternal separation on the acoustic startle reflex in rats: early baseline enhancement in females and blunted response to ambiguous threat
Source: Front Behav Neurosci. 2022 Oct 26;16:1023513. doi: 10.3389/fnbeh.2022.1023513 (PMC9643533; doi:10.3389/fnbeh.2022.1023513)
Supplement: Supplementary file 1 [file Data_Sheet_1.docx]

Supplementary Table 1: Results of 2-way ANOVAs for sex and rearing effects on acoustic startle outcome measures in the baseline test on P24, 34, and 54 in Experiment 1. Significant effects *p*<.05 are bolded.

| *Age* | *Dependent Variable* | *Comparison* | *F_(1,37)_* | *p* | *Effect size (η_p_^2^)* |
| --- | --- | --- | --- | --- | --- |
| P24 | Latency to Startle | Sex | 2.919 | 0.096 | 0.073 |
|  |  | Rearing | 2.192 | 0.147 | 0.056 |
|  |  | Interaction | 0.165 | 0.687 | 0.004 |
| P24 | Peak Time | **Sex** | **6.1045** | **0.018** | **0.142** |
|  |  | Rearing | 0.6523 | 0.424 | 0.017 |
|  |  | Interaction | 0.054 | 0.817 | 0.001 |
| P24 | Peak Value | Sex | 0.01946 | 0.89 | 0.001 |
|  |  | Rearing | 0.00851 | 0.927 | 0 |
|  |  | Interaction | 1.01631 | 0.32 | 0.027 |
| P24 | Duration | Sex | 2.67 | 0.111 | 0.067 |
|  |  | Rearing | 2.49 | 0.123 | 0.063 |
|  |  | Interaction | 1.59 | 0.215 | 0.041 |
| *Age* | *Dependent Variable* | *Comparison* | *F_(1,36)_* | *p* | *Effect size (η_p_^2^)* |
| P34 | Latency to Startle | Sex | 2.71 | 0.109 | 0.07 |
|  |  | Rearing | 4.05 | 0.052 | 0.101 |
|  |  | Interaction | 1.23 | 0.274 | 0.033 |
| P34 | Peak Time | Sex | 2.19 | 0.148 | 0.06 |
|  |  | Rearing | 2.96 | 0.094 | 0.076 |
|  |  | Interaction | 1.14 | 0.294 | 0.031 |
| P34 | Peak Value | Sex | 0.216 | 0.645 | 0.01 |
|  |  | **Rearing** | **12.476** | **0.001** | **0.257** |
|  |  | **Interaction** | **6.163** | **0.018** | **0.146** |
| P34 | Duration | Sex | 0.0331 | 0.857 | 0.00 |
|  |  | Rearing | 1.477 | 0.232 | 0.039 |
|  |  | Interaction | 0.5864 | 0.449 | 0.016 |
| *Age* | *Dependent Variable* | *Comparison* | *F_(1,35)_* | *p* | *Effect size (η_p_^2^)* |
| P54 | Latency to Startle | Sex | 3.407 | 0.073 | 0.089 |
|  |  | Rearing | 0.394 | 0.534 | 0.011 |
|  |  | **Interaction** | **5.734** | **0.022** | **0.141** |
| P54 | Peak Time | Sex | 1.459 | 0.235 | 0.04 |
|  |  | Rearing | 0.276 | 0.603 | 0.008 |
|  |  | **Interaction** | **5.304** | **0.027** | **0.132** |
| P54 | Peak Value | **Sex** | **9.394** | **0.004** | **0.212** |
|  |  | Rearing | 1.026 | 0.318 | 0.028 |
|  |  | Interaction | 0.304 | 0.585 | 0.009 |
| P54 | Duration | **Sex** | **23.1727** | **< .001** | **0.398** |
|  |  | Rearing | 0.0151 | 0.903 | 0 |
|  |  | Interaction | 0.8031 | 0.376 | 0.022 |

Supplementary Table 2: Results of 2-way ANOVAs for sex and rearing effects on the percent change in acoustic startle outcome measures after exposure to 22kHz ultrasonic vocalization compared to baseline responses at P25, 35, and 55 in Experiment 1. Significant effects *p*<.05 are bolded.

| *Age* | *Dependent Variable* | *Comparison* | *F_(1,37)_* | *p* | *Effect size (η_p_^2^)* |
| --- | --- | --- | --- | --- | --- |
| P25 | %Δ Latency to Startle | **Sex** | **4.66** | **0.037** | **0.112** |
|  |  | Rearing | 2.32 | 0.136 | 0.059 |
|  |  | Interaction | 2.24 | 0.143 | 0.057 |
| P25 | %Δ Peak Time | Sex | 2.92 | 0.096 | 0.075 |
|  |  | **Rearing** | **7.01** | **0.012** | **0.163** |
|  |  | Interaction | 2.03 | 0.163 | 0.053 |
| P25 | %Δ Peak Value | Sex | 0.247 | 0.622 | 0.007 |
|  |  | Rearing | 0.809 | 0.374 | 0.021 |
|  |  | Interaction | 0.231 | 0.634 | 0.006 |
| P25 | %Δ Duration | Sex | 0.03 | 0.863 | 0.001 |
|  |  | Rearing | 1.7377 | 0.196 | 0.045 |
|  |  | Interaction | 1.0434 | 0.314 | 0.027 |
| *Age* | *Dependent Variable* | *Comparison* | *F_(1,36)_* | *p* | *Effect size (η_p_^2^)* |
| P35 | %Δ Latency to Startle | Sex | 0.189 | 0.666 | 0.005 |
|  |  | Rearing | 2.495 | 0.123 | 0.065 |
|  |  | Interaction | 0.119 | 0.732 | 0.003 |
| P35 | %Δ Peak Time | Sex | 0.5647 | 0.457 | 0.015 |
|  |  | Rearing | 2.1283 | 0.153 | 0.056 |
|  |  | Interaction | 0.04 | 0.843 | 0.001 |
| P35 | %Δ Peak Value | Sex | 0.025 | 0.875 | 0.001 |
|  |  | Rearing | 0.7846 | 0.382 | 0.021 |
|  |  | Interaction | 0.0134 | 0.909 | 0.000 |
| P35 | %Δ Duration | Sex | 0.4637 | 0.5 | 0.013 |
|  |  | Rearing | 0.1534 | 0.698 | 0.004 |
|  |  | Interaction | 0.0104 | 0.919 | 0.000 |
| *Age* | *Dependent Variable* | *Comparison* | *F_(1,37)_* | *p* | *Effect size (η_p_^2^)* |
| P55 | %Δ Latency to Startle | Sex | 0.5468 | 0.465 | 0.015 |
|  |  | Rearing | 0.013 | 0.91 | 0 |
|  |  | Interaction | 0.2233 | 0.639 | 0.006 |
| P55 | %Δ Peak Time | Sex | 0.498 | 0.485 | 0.014 |
|  |  | Rearing | 0.072 | 0.79 | 0.002 |
|  |  | Interaction | 0.504 | 0.482 | 0.014 |
| P55 | %Δ Peak Value | **Sex** | **7.469** | **0.01** | **0.176** |
|  |  | **Rearing** | **4.127** | **0.05** | **0.105** |
|  |  | Interaction | 0.588 | 0.448 | 0.017 |
| P55 | %Δ Duration | Sex | 0.142 | 0.709 | 0.004 |
|  |  | Rearing | 0.511 | 0.48 | 0.014 |
|  |  | Interaction | 2.679 | 0.111 | 0.071 |

Supplementary Table 3: Results of 2-way ANOVAs for sex and rearing effects on acoustic startle outcome measures in the baseline test on P54 in Experiment 2. Significant effects *p*<.05 are bolded.

| *Age* | *Dependent Variable* | *Comparison* | *F_(1,36)_* | *p* | *Effect size (η_p_^2^)* |
| --- | --- | --- | --- | --- | --- |
| P54 | Latency to Startle | Sex | 1.756 | 0.193 | 0.047 |
|  |  | Rearing | 2.74e-4 | 0.987 | 0.000 |
|  |  | Interaction | 0.539 | 0.468 | 0.015 |
| P54 | Peak Time | Sex | 0.196 | 0.660 | 0.005 |
|  |  | Rearing | 0.170 | 0.682 | 0.005 |
|  |  | Interaction | 0.574 | 0.453 | 0.016 |
| P54 | Peak Value | Sex | 2.241 | 0.143 | 0.059 |
|  |  | Rearing | 0.251 | 0.619 | 0.007 |
|  |  | Interaction | 4.01e-4 | 0.984 | 0.000 |
| P54 | Duration | **Sex** | **51.801** | **<.001** | **0.590** |
|  |  | **Rearing** | **5.025** | **0.031** | **0.122** |
|  |  | Interaction | 0.264 | 0.610 | 0.007 |

Supplementary Table 4: Results of 2-way ANOVAs for sex and rearing effects on the percent change in acoustic startle outcome measures during exposure to light compared to baseline responses at P55 in Experiment 2.

| *Age* | *Dependent Variable* | *Comparison* | *F_(1,36)_* | *p* | *Effect size (η_p_^2^)* |  |
| --- | --- | --- | --- | --- | --- | --- |
| P55 | %Δ Latency to Startle | Sex | 1.050 | 0.312 | 0.028 |  |
|  |  | Rearing | 2.199 | 0.147 | 0.058 |  |
|  |  | Interaction | 0.166 | 0.686 | 0.005 |  |
| P55 | %Δ Peak Time | Sex | 2.04 | 0.162 | 0.054 |  |
|  |  | Rearing | 2.06 | 0.160 | 0.054 |  |
|  |  | Interaction | 6.54e-4 | 0.980 | 0.000 |  |
| P55 | %Δ Peak Value | Sex | 0.044 | 0.835 | 0.001 |  |
|  |  | Rearing | 2.810 | 0.102 | 0.072 |  |
|  |  | Interaction | 0.0248 | 0.876 | 0.001 |  |
| P55 | %Δ Duration | Sex | 0.014 | 0.906 | 0.000 |  |
|  |  | Rearing | 0.0186 | 0.892 | 0.001 |  |
|  |  | Interaction | 0.0167 | 0.898 | 0.000 |  |

Supplementary Table 5: Results of repeated measures (RM) ANOVA on peak startle in the light-enhanced startle test at P55 and post-hoc test comparing the test at baseline (no light) to test during light exposure (light) in control (Con) and maternal separation (MS) rats. Significant effects *p*<.05 are bolded.

| *Dependent Variable* | *Comparison* | *F* | *p* | *Effect size (η_p_^2^)* |
| --- | --- | --- | --- | --- |
| Peak Value | **Light exposure** | **6.09583** | **0.018** | **0.145** |
|  | Light*Sex | 0.433 | 0.515 | 0.012 |
|  | **Light*Rearing** | **4.943** | **0.033** | **0.121** |
|  | Light*Sex*rearing | 0.004 | 0.952 | 0.000 |

| RM Factor | Rearing |  | RM Factor | Rearing | t | p_tukey_ |
| --- | --- | --- | --- | --- | --- | --- |
| **No light** | **Con** | - | **Light** | **Con** | **-3.405** | **0.009** |
| No light | Con | - | Light | MS | 0.427 | 0.973 |
| No light | MS | - | Light | Con | -2.443 | 0.087 |
| No light | MS | - | Light | MS | -0.169 | 0.998 |
| Light | Con | - | Light | MS | 2.483 | 0.080 |
